# Anticarcinogenic and Antioxidant Action of an Edible Aquatic Flora *Jussiaea repens* L. Using In Vitro Bioassays and In Vivo Zebrafish Model

**DOI:** 10.3390/molecules26082291

**Published:** 2021-04-15

**Authors:** Chongtham Rajiv, Subhra Saikat Roy, K. Tamreihao, Pintubala Kshetri, Thangjam Surchandra Singh, Haobijam Sanjita Devi, Susheel Kumar Sharma, Meraj Alam Ansari, Elangbam Diana Devi, Asem Kajal Devi, Pangambam Langamba, Heikham Naresh Singh, Romila Akoijam, Chongtham Tania, Chongtham Sonia

**Affiliations:** 1 ICAR-NEH Quality Analysis Laboratory, ICAR Research Complex for NEH Region, Manipur Centre, Imphal 795004, India; rajivchongtham@yahoo.com (C.R.); tammasi2009@gmail.com (K.T.); pintuksh@gmail.com (P.K.); surthangjam@gmail.com (T.S.S.); susheel.sharma@icar.gov.in (S.K.S.); meraj.ansari@icar.gov.in (M.A.A.); asemkajal@gmail.com (A.K.D.); langambakhuman007@gmail.com (P.L.); naresheikham@gmail.com (H.N.S.); romila.akoijam@icar.gov.in (R.A.); chongtham.tania@icar.gov.in (C.T.); chongtham.sonia@icar.gov.in (C.S.); 2 College of Horticulture, Central Agricultural University, Sikkim 737135, India; sanjita.cohb@cau.ac.in; 3 Department of Biochemistry, Manipur University, Imphal 795003, India; dianadevi33@gmail.com

**Keywords:** *Jussiaea repens* L., antioxidant activity, antiproliferative properties, anticancer, SKOV3, HeLa, MDA-MB-231, PANC-1, PC3, zebrafish

## Abstract

Oxidative stress is the major cause of many health conditions, and regular consumption of antioxidants helped to encounter and prevent such oxidative stress-related diseases. Due to safety concerns over long-term uses of synthetic antioxidants, natural antioxidants are more preferred. The purpose of this study is to investigate the antioxidant and anticancer activities of *Jussiaea repens* L., a wild edible flora found in Manipur, India. The antioxidant activity was evaluated using 1,1-diphenyl-2-picrylhydrazyl (DPPH), 2,2′-azino-bis (3-ethylbenzothiazoline-6-sulfonic acid) (ABTS), Ferric reducing antioxidant power (FRAP) assay and DNA-nicking assay. The anticancer activity was tested using five cancer lines viz., SKOV3 cells (ovarian), HeLa (cervical), MDA-MB-231 (breast), PANC-1 (pancreatic), and PC3 (prostate). The toxicity, developmental effect, antiproliferative activity was further tested using zebrafish embryos. The methanolic plant extract had higher polyphenol content than flavonoids. The in vitro study demonstrated a promising antioxidant capacity and DNA protection ability of this plant. The extract also showed cytotoxic activity against SKOV3, HeLa, MDA-MB-23, and PANC-1 cancer cell lines. The in vivo studies on zebrafish embryos demonstrated the extract’s ability to suppress the developmental process and elicited more cytotoxicity to cancer cells than developing zebrafish embryos. Moreover, the in vivo studies on zebrafish embryos also indicated the antiproliferative activity of *J. repens* L. extract.

## 1. Introduction

Oxidative stress is the major cause of many health conditions, such as diabetes, cancer, cardiovascular diseases, aging and neurodegenerative disease like Alzheimer’s [1,2,3,4]. This adverse effect is due to disruption in the balance between the formation of reactive oxygen species (ROS) and the body’s mechanism to counter such elevated reactive species [5]. Nevertheless, antioxidant reduces the oxidative damage by directly scavenging the ROS, chelating, or elevating cellular antioxidant enzyme activities, such as glutathione reductase, catalase and superoxide dismutase that mitigate the oxidative stress [6]. Regular consumption of antioxidants as a part of nutraceuticals or dietary supplements at an appropriate dose helped to encounter and prevent the body from oxidative stress-related diseases [6]. Due to safety issues over long-term uses of synthetic antioxidants, the natural origin antioxidants are more preferred to formulate nutraceuticals and dietary supplements [7,8]. Therefore, identifying plants with potential antioxidant activity will significantly impact human health and economic benefit.

Mankind has been exploring plants as a source of bioactive phytochemicals to treat various kinds of diseases since time immemorial. Numerous studies have been done to determine the medicinal properties of plants used by ethnic people for treating various diseases. Since plants are rich in bioactive compounds, there is a great prospect to discover new compounds or drugs having health-beneficial properties. Many phenolic and flavonoid compounds exhibiting beneficial health activities, such as antioxidant, anti-inflammatory, anticancer, antihypertensive and anti-obesity, have been identified from the ethnomedicinal plants [8,9,10].

Additionally, more than 60% of anticancer compounds identified were directly or indirectly extracted from plants [9]. However, most of the anticancer compounds have cytotoxic effects on the tumor cells and the normal cells. Hence, the discovery of potent bioactive compounds having cytotoxicity towards cancer cells and low toxicity to normal cells from the plants used in folk medicine is of high significance [11].

For in vivo cytotoxicity assay, Zebrafish (*Danio rerio*) has emerged as a powerful vertebrate model in the field of drug discovery for the rapid study of the mechanism of action test compounds. Many notable characteristics of zebrafish making it a good model are due to its high fecundity, small size, rapid developmental process and optical transparency during the early stages of development [12,13,14]. Moreover, due to the transparent nature of the embryo and comparable embryogenesis process with cancer cells, the zebrafish embryo is being used for a rapid and cost-efficient in vivo model for screening of anticancer potential of a compound [15].

*Jussiaea repens* L. (Family: Onagraceae), commonly known as water primrose, is an edible plant mostly found in the aquatic ecosystem. The plant is consumed as vegetables and used to treat certain diseases by ethnic people. However, a meager report is available on the beneficial health properties of this plant.

This prompted us to study the *J. repens* L. extract for its antioxidant and anticancer activities under in vitro conditions using five different cancer cell lines and further validate the anticancer activity through in vivo assay using the zebrafish model.

## 2. Results

### 2.1. Total Polyphenol and Flavonoid Content

The total polyphenol and flavonoid content of *J. repens* L. were expressed as gallic acid equivalent (GAE/g extract weight) and quercetin equivalent (QE/g extract weight), respectively. The polyphenol content was found to be much higher than flavonoids, as expected (Table 1). The polyphenol and flavonoid content of the plant extract was found to be 193.01 mg GAE/g ext. wt. and 2.75 mg QE/g ext. wt., respectively (Table 1).

### 2.2. In Vitro Antioxidant Assay

The crude extract of *J. repens* L. demonstrated potential antioxidant activities. The antioxidant activity of the plant extract in different assays was compared concerning ascorbic acid. The maximum antioxidant activity was recorded in FRAP, followed by ABTS*^+^ and DPPH^•^ assays. The IC_50_ of ABTS*^+^ free radical scavenging activity of the crude extract was found to be 211.55 µg/mL, and that of ascorbic acid was found to be 44.47 ± 1.63 µg/mL (Table 1). Similarly, the IC_50_ values of DPPH^•^ free radicals scavenging activity of the crude extract and the ascorbic acid were 439.36 µg/mL and 63.41 µg/mL, respectively (Table 1), whereas the Fe^3+^ reducing antioxidant power of the extract and ascorbic acid was 24.48 mM and 72.57 mM equivalent of FeSO_4_, respectively (Table 1).

### 2.3. DNA Nicking Assay

**Figure 1:**
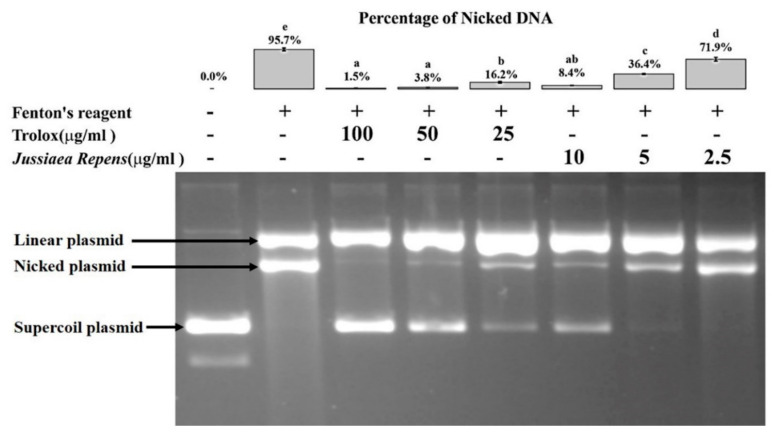
In vitro DNA protection capacity of *Jussiaea repens* L. The in vitro DNA protection capacity of *J. repens* L. from hydroxyl radicals. The bar graph represents the percentage (Mean ± SEM) of nicked from plasmid produced due to Fenton’s reagent. The values with the same alphabet are not statistically significant at a 5% level of significance according to Tukey’s HSD test.

The assay involved analyzing the DNA protection ability of a compound against hydroxyl radicals generated by Fenton’s reagent. The cleavage of pUC19 plasmid DNA by Fenton’s reagent led to the formation of linear and nicked DNA from the supercoiled double-stranded form of DNA. Out of these two cleaved plasmid DNA forms, the nicked form represents the degraded plasmid DNA due to the action of •OH radical, while the other form is due to Fe^2+^ [16]. Therefore, the amount of nicked form present in the reactions was used to assess the test sample’s DNA protection ability. The amount of nicked form present in the reaction treated with Fenton’s reagent only was considered as 100% cleavage by •OH radical. In our study, *J. repens* L. extract significantly reduced the percentage of nicked plasmid DNA formation, indicating the capability to protect DNA damage caused by hydroxyl free radicals. The percentage of nick plasmid formation was further reduced with an increase in the plant extract concentration. The treatment of 10 µg/mL of the plant extract exhibited 8.5% plasmid degradation, which was statistically at par with a much higher dose of positive control Trolox (25 µg/mL), displaying 16.2% plasmid degradation (Figure 1).

### 2.4. In Vitro Cytotoxicity Assay

*J. repens* L. extract showed significant cytotoxic effect against SKOV3 (ovarian), HeLa (cervical), MDA-MB-23 (breast) and PANC-1 (pancreatic) cancer cell lines (Table 2). Among the four cancer cell lines, maximum anticancer activity was recorded with MDA-MB (EC_50_ 46.13 ± 1.94 µg/mL), followed by SKOV3 (56.26 ± 5.65 µg/mL), HeLa (61.98 ± 5.71 µg/mL) and Panc-1 (71.1 ± 5.03 µg/mL). However, no cytotoxic activity was observed against PC-3 (prostate cancer cells).

### 2.5. In Vivo Cytotoxicity Assay

**Figure 2:**
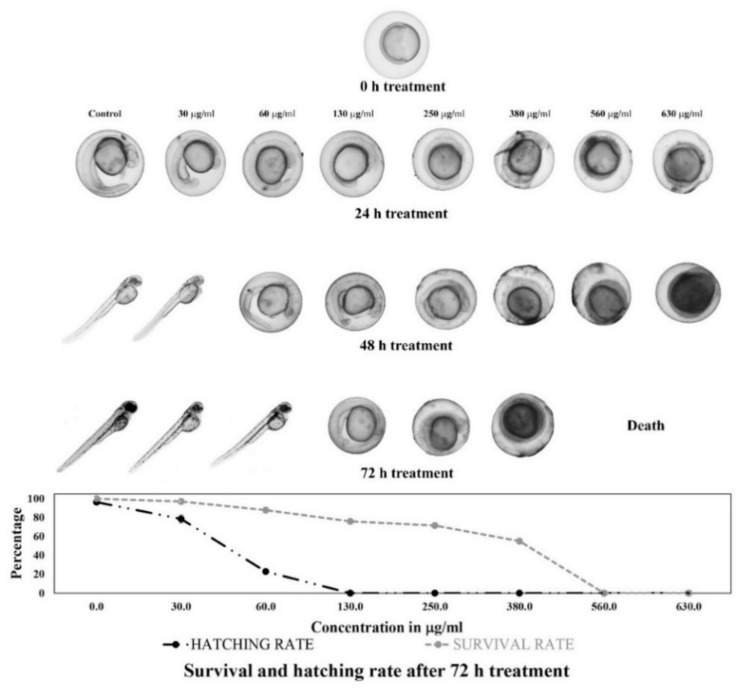
In vivo developmental effect of *Jussiaea repens* L. The developmental effect of *J. repens* L. in the developing embryo of zebrafish (0–6 hpf) was studied by treating the embryo with different plant extract concentrations. The line graph represents the hatching rate and survival rate.

The 6 h post-fertilization (hpf) developing embryos showed variation in developmental stages when treated with different plant extract concentrations (30–630 µg/mL). Embryos treated with 30 µg/mL showed the survival rate, hatching rate (started from 48 h (h)) and the developmental process almost comparable to the control group. However, treatment with 60 µg/mL of plant extract delayed the developmental process. Further increase in the concentration (130–380 µg/mL) resulted in underdeveloped embryos, and the embryos were not able to hatch even after 72 h. Moreover, the embryos could not survive at 560 µg/mL concentration (Figure 2). The cytotoxicity study of the plant extract against 72 hpf zebrafish embryo revealed that the plant extract had an LC_50_ value of 169.2 ± 1.3 µg/mL.

**Figure 3:**
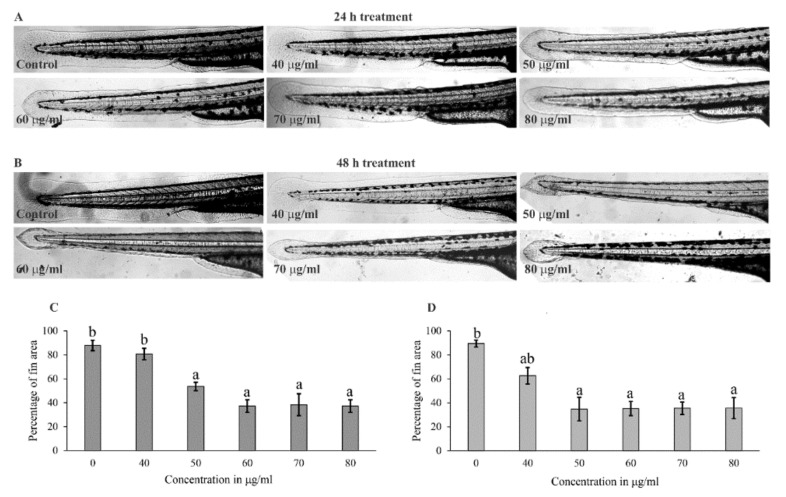
In vitro fin reduction assay after *Jussiaea repens* L. treatment. The reduction in the fin area of 72 hpf zebrafish embryo after treatment of plant extract at 24 h posttreatment (**A**) and 48 h posttreatment (**B**). The bar graph represents the percentage reduction (mean **±** SEM) of a fin area after 24 h posttreatment (**C**) and 48 h posttreatment (**D**) of *J. repens* L. extract. The values with the same alphabet are not statistically significant at a 5% level of significance according to Tukey’s HSD test.

The antiproliferative activity of *J. repens* L. extract was also studied using 72 hpf zebrafish embryos. There was no change observed in fin morphology at a concentration of 40 µg/mL after 24 h posttreatment than the control group (Figure 3A,C). In contrast, a slight reduction in the fin area was observed after 48 h posttreatment (Figure 3B,D). However, the fin area was reduced significantly at a concentration of 50 µg/mL after 24 h posttreatment, and no further reduction in fin area was noticed beyond 50 µg/mL (Figure 3A,C). Similar results were also observed for 48 h posttreatment (Figure 3B,D).

## 3. Discussion

Plants are well known as a potential source of bioactive compounds, and such phyto-origin compounds are more preferred over synthetic ones [8]. Hence, researchers are looking for neglected or underutilized medicinally important edible plants to discover new bioactive compounds. The present study on the methanolic extract of *J. repens* L., an underutilized aquatic plant of North East Indian Himalaya, revealed potential anticancer activity with minimal toxicity to the normal cell. The plant has been previously reported to exhibit in vitro antioxidant and hepatoprotective activity under in vivo study by enhancing the cellular antioxidant system in the liver [17,18]. The anticancer activity of *J. repens* L. extract against breast cancer (Ehrlich Ascites) was studied by Marzouk et al. [18]. The major components for elucidating the bioactivity of this plant, particularly flavonoids, have also been reported [17,18]. As the earlier study was limited to breast cancer cell lines, the effectiveness of this plant extract against other important cancer cells is still missing. Furthermore, depending on habitat and environmental conditions, the bioactive property of the same plant species may vary [19]. Hence, the present study on *J. repens* L. endemic to North East Indian Himalaya represents the first report on its anticancer activity.

In the present study, *J. repens* L. was found to possess higher polyphenol content than the flavonoids. Singh et al. [20] also reported that the acetone extract of *J. repens* L had higher polyphenol content than the flavonoids. The potent antioxidant and anticancer activities of the plant extract may be attributed to its high polyphenol content. Other researchers also reported a strong correlation of high polyphenol content with beneficial health properties, such as antioxidant, anticancer, anti-inflammatory and antidiabetic activities [8,21,22,23].

The oxidative damage caused in the DNA level is one of the important factors for developing many pathological conditions [24]. Antioxidant compounds extracted from the plant can be used to prevent such oxidative damage for healthy living. The DNA protection capacity of a compound under in vitro conditions is often evaluated using the DNA nicking assay, which mimics the in vivo condition. Fenton’s reagent-based method is the most popular approach as it simulates the biological system of intracellular iron-dependent hydroxyl radical’s production [16]. *J. repens* L. showed a promising result on DNA protective capability even more than Trolox. The methanolic plant extract (10 µg/mL) could protect 91.5% plasmid DNA against free radicals compared to 83.8% by Trolox (25 µg/mL). This indicates that *J. repens* L. is endowed with commercially exploitable antiradical activity.

*J. repens* L. extract also demonstrated anticancer activities against four cancer cell lines, namely, SKOV3, HeLa, MDA-MB-231 and PANC-1. The extract showed maximum anticancer activity against MDA-MB-231 (EC_50_ 46.13 µg/mL), followed by SKOV3 (EC_50_ 56.26 µg/mL), HeLa (EC_50_ 61.98 µg/mL) and Panc-1 (EC_50_ 71.1 µg/mL) cell lines.

The in vitro cancer cell proliferation inhibitory activity of the plant extract has been further validated using zebrafish embryos. Our study also showed that *J. repens* L. had effective antiproliferative activity and can be further explored for its pharmaceutical application. The treatment of *J. repens* L. extract on the developing zebrafish embryo delayed the developmental process in the dose-dependent manner as indicated by a reduction in hatching rate and slowing down the developmental stages. In addition, fin reduction assay using zebrafish embryo, another model for the rapid screening of compounds having an antiproliferative activity [14,25,26], was done. The changes in fin morphology due to apoptosis after the treatment with plant extract suggested the antiproliferative property of *J. repens* L. At a 50 µg/mL concentration, a significant reduction (around 40%) in the fin area of the zebrafish embryo was observed after 24 h of treatment. Further increase in the concentration of plant extract did not reduce the fin area. A slight reduction in fin area was observed when treated with a 40 µg/mL of extract after 48 h posttreatment. Similarly, no significant difference was observed among the treatments of 50, 60, 70 and 80 µg/mL of extract even after 48 h posttreatment. Among the different parts of the fin, reduction in the dorsal fin area was more prominent.

The majority of the anticancer drugs showed toxicity to tumor cells and normal healthy cells, which induces severe adverse side effects to human health. Therefore, the effectiveness of an anticancer drug not only depends on its cytotoxicity towards cancer cells but also demands a minimal toxic effect on the normal cells [11]. As the morphogenesis and development of the primary organ system of zebrafish embryos complete in 72 hpf, this stage of the embryo are frequently used in the in vivo cytotoxicity study. The LC_50_ value of the *J. repens* L. extract (169.2 ± 1.3 µg/mL) in the in vivo assay was found to be more than twice the highest EC_50_ value of the tested cell lines, i.e., Panc-1 (71.1 ± 5.03 µg/mL) in the in vitro cytotoxicity assay, indicating potential cytotoxicity of the plant extract on cancer cells, while low toxicity on the normal cells. Hence, the antioxidant and anticancer activity of *J. repens* L. deciphered in the present study suggests that this underutilized aquatic plant is a potential candidate for developing anticancer drugs.

## 4. Materials and Methods

### 4.1. Sample Collection and Extraction

The plant sample (*J. repens* L.) was collected from the Experimental Farm of ICAR Research Complex for NEH Region, Manipur Centre, Imphal (24.8284° N, 93.9260° E, at 774 m above mean sea level), maintained under a hydroponic system. The plant sample was washed properly with distilled water and allowed to dry at 40 °C in a hot-air oven. The dried samples were minced into powder form and stored in a glass vial at 4 °C until further use. A 100 g of powdered sample was mixed with 500 mL methanol and kept in an orbital rotary shaker (New Brunswick Scientific, Humburg, Germany) at 150 rpm for 6 h at room temperature (RT). The homogenate was centrifuged (10,000 rpm for 10 min at 4 °C), and the supernatant obtained through centrifugation was allowed to pass through Whatman filter paper (no. 1). The crude extract was dried under reduced pressure using a rotatory vacuum evaporator (IKA, Staufen, Germany) and stored at −20 °C until further use. Before analyzing, the samples were dissolved in 50% DMSO. The final working concentration of all the assays was performed at 0.25% DMSO.

### 4.2. Estimation of Total Polyphenol and Flavonoid Content

The estimation of total polyphenol and flavonoid content was performed in triplicates for each concentration.

#### 4.2.1. Total Flavonoid Contents

The total flavonoid content of *J. repens* L. extract was measured according to the aluminum chloride (AlCl_3_) colorimetric method [27]. The reaction mixture was prepared by mixing 300 µL of the sample (containing 5 to 150 µg), 300 µL of 2% AlCl_3_. The absorbance was measured at 420 nm using a UV-vis spectrophotometer (Merck, Germany) after 1 h of incubation at RT. The total flavonoid content of the sample was calculated from a standard curve of quercetin (0.10–3.0 µg, r^2^ = 0.999) and expressed in mg of quercetin equivalent (QE) per gram of extract weight (QE/g ext. wt).

#### 4.2.2. Total Polyphenol Contents

The total polyphenol content was estimated as Folin–Ciocâlteu (FC) reducing capacity. In this assay, 200 µL of the sample (containing 4 to 40 µg) was mixed with 200 µL of 0.2 N Folin–Ciocâlteu reagent and kept for 5 min at RT. A 160 µL of 8% sodium carbonate (Na_2_CO_3_) was then added and incubated for 2 h at 25 °C. The absorbance was measured at 750 nm against the reagent blank. The amount of total polyphenol content was calculated from a standard curve of gallic acid (0.20–2.0 μg, r^2^ = 0.998) and expressed in mg of gallic acid equivalent (GE) per gram of extract weight (GAE/g ext. wt).

### 4.3. In Vitro Antioxidant Assays

All in vitro antioxidant activity was measured in triplicate for each concentration.

#### 4.3.1. DPPH Radical Scavenging Assay

The methanolic extract of *J. repens* L. was screened for free radical scavenging activity using a 1,1-diphenyl-2-picrylhydrazyl (DPPH) assay with ascorbic acid as a positive control. Briefly, 1 mM/L DPPH solution was prepared in 100 percent methanol. From this, 950 µL of DPPH solution was then individually mixed with 50 µL of the sample (62.50–625.00 µg/mL) and ascorbic acid (10–100 µg/mL). The solutions were then incubated in dark conditions at 37 °C for 30 min. The absorbance was measured at 517 nm. The fading of the deep violet color of the DPPH solution was used to measure the strength of antioxidant capacity. The percentage of DPPH free radical scavenging was calculated using the following formula:

(1) $${\mathrm{Percentage}\;\mathrm{scavenging}\;\mathrm{of}\;\mathrm{DPPH}}^{•}=\frac{\left(A_{0}-A_{1}\right)}{A_{0}}\;X\;100$$

where

A_0_ = absorbance of the blank;
A_1_ = absorbance given by sample/ascorbic acid.

#### 4.3.2. ABTS Radical Scavenging Assay

2,2′-azino-bis (3-ethylbenzothiazoline-6-sulfonic acid) free radicals (ABTS*^+^) scavenging activity of plant extract was evaluated following the prescribed protocol [28]. ABTS*^+^ solution containing 7.5 mM ABTS and 2.6 mM potassium persulfate (K_2_S_2_O_8_) was prepared in ethanol and incubated for 12 h at RT to generate free radicals. Before performing the assay, the ABTS*^+^ solution was diluted with ethanol to get an absorbance of around 0.8 ± 0.1. The reaction mixture was prepared by mixing 950 µL of ABTS solution and 50 µL of plant sample (89.31–230.41 µg/mL) or ascorbic acid (10–100 µg/mL). The reaction mixtures were then incubated for 30 min under dark conditions at RT. The absorbance changes were estimated using a UV-vis spectrophotometer (Merck, Darmstadt, Germany) at 734 nm.

(2) $${\mathrm{Percentage}\;\mathrm{scavenging}\;\mathrm{of}\;\mathrm{ABTS}}^{*+}=\frac{\left(A_{0}-A_{1}\right)}{A_{0}}\;X\;100$$

where

A_0_ = absorbance of the blank;
A_1_ = absorbance given by sample/ascorbic acid.

#### 4.3.3. Ferric (Fe^3+^) Reducing Antioxidant Power Assay (FRAP)

FRAP assay was done according to Benzie and Strain [29]. The FRAP reagent was prepared by mixing 0.3 M acetate buffer (pH 3.6), 10 mM TPZ (2,4,6-Tri-(2-pyridyl)-5-triazine in 40 mM HCl) and 20 mM FeCl_3_ in the ratio of 10:1:1. The FRAP reagent was then incubated at 37 °C for 30 min. The reaction mixture was prepared by mixing 950 µL of FRAP reagent with 50 µL of the sample. The calibration curve of FeSO_4_ containing 5–100 nM was prepared to calculate the FRAP value and the antioxidant power interpreted as mM equivalent of FeSO_4_. The intense blue color developed was then read after 5 min using a UV-vis spectrophotometer (Merck, Germany) at 593 nm.

(3) $${\mathrm{FRAP}\;\mathrm{value}\;\mathrm{or}\;\mathrm{mM}\;\mathrm{Ferrous}\;\mathrm{Equivalents}}^{\;}=\frac{D}{V}\;X\;F$$

where

D = sample dilution factor of the sample to fit within the standard curve range;
V = sample volume in μL;
F = ferrous amount from the standard curve (nmol).

### 4.4. DNA Nicking Assay

The DNA nicking assay was performed using Fenton’s reagent [30]. The reaction mixture was prepared in triplicates by combining 5 µL Fenton’s reagent (41.5 mM pH 7.4 phosphate buffer, 0.2 mM FeSO_4_, 980 mM H_2_O_2_), 0.5 µL pUC19 plasmid (1 µg/µL) and 4.5 µL of extract. Trolox (6-Hydroxy-2,5,7,8-tetramethylchromane-2-carboxylic acid) and water were used as a positive and negative control, respectively. The concentrations of the extract added per reaction were 2.5, 5.0 and 10.0 µg/mL, and that of Trolox were 25, 50 and 100 µg/mL. The reaction mixtures were incubated at 37 °C for 30 min to allow the degradation of plasmid DNA by Fenton’s reagent. The above reaction mixtures were run at 1.5% agarose gel containing ETBR in TAE buffer (120 min at 50 V) to analyze the degree of DNA degradation. The gel image was visualized using a gel imaging system (ChemiDoc XRS+, Bio-Rad, CA, USA). The amount of nicked forms of pUC19 plasmid DNA was quantified using Image Lab 5.2.1 (Bio-Rad, CA, USA).

### 4.5. In Vitro Cytotoxicity Assay

The cytotoxic activity of *J. repens* L. extract was investigated using sulforhodamine B colorimetric (SRB) assay against five cancer lines viz., human ovarian cancer cell line (SKOV3 cells), human cervical cancer cell line (HeLa), human breast cell line (MDA-MB-231), human pancreatic cancer cell line (PANC-1), and human prostate cancer cells (PC3). The SRB staining is not cell-line dependent and hence, provides better sensitivity. Moreover, in contrast to other colorimetric assays, the SRB dye stains only recently lysed cells, not the cell debris [31]. The cancer cells were maintained in a Dulbecco’s modified Eagle’s medium (DMEM), minimal essential medium (MEM), Roswell Park Memorial Institute (RPMI), supplemented with glutamine. The cells were subjected to different concentrations of *J. repens* L. extract (5–100 µg/mL) in triplicates for 48 h. The known cancer drug doxorubicin (5–100 µg/mL) was used as a positive control. The monolayer cells were then fixed with 10% trichloroacetic acid (TCA) for 1 h at 40 °C followed by washing with water. After drying, the cell plates were stained with SRB for 30 min and then washed with 1% acetic acid. The SRB dye bound to protein was then dissolved by adding 10 mM Tris base (pH 10.5). The SRB dye released was quantified by taking absorbance at 510 nm in Varioskan flash multimode reader (Thermo Fisher Scientific, MA, USA). The EC_50_ (effective concentration) value of the plant extract and positive control against each cell line were determined as described by Vichai and Kirtikara [32].

### 4.6. In Vivo Cytotoxicity Assay Using Zebrafish Embryo

#### 4.6.1. Zebrafish Rearing and Housing Conditions

The experiment performed using zebrafish in the study was approved by the Institute Animal Ethics Committee of ICAR Research Complex for NEH Region (registration no. 2100/GO/RBi/L/20/CPCSEA dated 29/05/20) vide IEC approval no. RC/IEC/2020/15. The experiments were performed according to CPCSEA guidelines, EU Directive 2010/63/EU and OECD [33].

The zebrafish (*Danio rerio*) reared was according to previously described protocols [34] with slight modifications. Both sexes of wild-type zebrafish (*Danio rerio*) were maintained under a lighting condition of 12 h light/ dark and water temperature of 28 °C. The water was continuously circulated through a biological filter at the rate of 1600 L/h except during feeding time (5–10 min, twice daily). The fishes were fed twice with commercial fish feed.

#### 4.6.2. Embryo Collection

The breeding of zebrafish was performed using gravid females and males of more than six months old by keeping the female and male in the breeding chamber at the ratio of 2:1 after a few evening feed hours. The spawning happened approximately within 1 h of light exposure in the morning. The adult fishes were cautiously taken out, the embryos were carefully collected and kept at 1X E3 embryo medium (60X E3 contain 5 mM NaCl, 0.17 mM, KCl, 10 mM HEPES, 0.33 mM MgSO_4_•7H_2_O, 0.33 mM CaCl_2_•6H_2_O) in the incubator at 28 °C until further use.

#### 4.6.3. In Vivo Screening of the Plant Extract for its Cytotoxic Effect on Embryo Development and Fin Morphology of Zebrafish

The embryos of 5–6 h post-fertilization (hpf) were treated with a different titer of extract (30, 60, 130, 250, 380, 560 and 630 µg/mL) to study the effect of plant extract on developing embryo. The developmental stages were then monitored using an optical microscope (Olympus, Tokyo, Japan) after 0, 24, 48 and 74 h of treatment. Experiments were performed in triplicates with 20 embryos for each treatment and the control group with 0.25% DMSO.

Using 72 hpf-hatched embryos, the mortality rate was analyzed using a nonlinear regression curve after 24 h of treatment to calculate the LC_50_ value (lethal concentration causing 50% mortality) of the plant extract. A concentration ranges from 156 to 321 µg/mL of extract was used in this study.

The antiproliferative activity of plant extract was further assessed by analyzing the fin morphology [14,25]. For this, the hatched embryo of 72 hpf was treated with 40, 50, 60, 70, and 80 µg/mL of *J. repens* L. extract, and the image captured using an optical microscope after 24 h and 48 h of incubation. The percentage reduction in the fin area of the zebrafish embryos was compared with the control. The highest fin areas in control were taken as 100%, and the result was illustrated by comparing with the treated embryo fins using Image J software (NIH, Bethesda, MD, USA). All the samples and the control were prepared to obtain a final DMSO concentration of 0.25% and 1X E3 medium. The experiment was performed in triplicates in 12 well cell culture plates containing 20 embryos per well.

### 4.7. Statistical Analysis

One-way ANOVA followed by Tukey HSD test was performed to analyze the significant variation (*p*-value ≤ 0.05) between the data using the Statgraphics Centurion XVI (Statpoint, Warrenton, VA, USA). The LC_50_ and EC_50_ values of the plant extract were determined using the Prism software (GraphPad, USA).

## 5. Conclusions

The present findings revealed the high polyphenol content and potent antioxidant capacity of *J. repens* L. This plant also showed the potential to protect the DNA from hydroxyl radicals and prevent the onset of oxidative stress-induced degenerative diseases. To the best of our knowledge, this is the first worldwide report on the anticancer activity of *J. repens* L. extract against ovarian, cervical and pancreatic cancer. Interestingly, our study on the zebrafish embryo also revealed a high cytotoxic effect on cancer cell-line and low toxicity to normal cells. The experimental results indicate that *J. repens* L. is a potential plant for the discovery of new promising anticancer drugs and antioxidant compounds that will subsequently have a substantial economic impact on pharmaceutical and nutraceutical industries. Further research to identify the bioactive compounds and underlying molecular mechanisms of cytotoxicity will provide first-hand knowledge of this plant in therapeutic applications for healthy living.

## Figures and Tables

**Table 1:** Polyphenols, flavonoids and in vitro antioxidant capacity of *J. repens* L.

Parameters	*J. repens* L. (Mean ± SEM)	Ascorbic Acid (Mean ± SEM)
Flavonoids (mg QE/g ext. wt.)	2.75 ± 0.11	--
Polyphenols (mg GAE/g ext. wt.)	193.01 ± 4.16	--
IC_50_ of DPPH assay (µg/mL)	439.36 ± 19.21	63.41 ± 3.20
IC_50_ of ABTS assay (µg/mL)	211.55 ± 21.19	44.47 ± 1.63
FRAP value * of 10 mg/mL extract	24.48 ± 0.77	72.57 ± 2.94

* FRAP value indicate mM equivalent of FeSO_4_.

**Table 2:** Cytotoxic effect of *J. repens* L. extracts on cancer cell lines.

	EC_50_ (µg/mL)				
	SKOV3	HeLa	MDA-MB-231	Panc-1	PC-3
*J. repens* L.	56.26 ± 5.65	61.98 ± 5.71	46.13 ± 1.94	71.1 ± 5.03	No toxicity
Doxorubicin	1.28 ± 0.34	2.37 ± 0.1	2.86 ± 0.95	2.09 ± 0.73	3.59 ± 0.43

SKOV3: human ovarian cancer cell line, HeLa: human cervical cancer cell line, MDA: MB-231-human breast cell line, PANC-1: human pancreatic cancer cell line, PC3: human prostate cancer cells.

## Data Availability

Data is contained within the article.

## Abbreviations

DPPH, 1,1-diphenyl-2-picrylhydrazyl; ABTS, 2,2′-azino-bis (3-ethylbenzothiazoline-6-sulfonic acid); FRAP, Ferric reducing antioxidant power; SKOV3, human ovarian cancer cell line; HeLa, human cervical cancer cell line; MDA-MB-231, human breast cell line; PANC-1, human pancreatic cancer cell line; PC3, human prostate cancer cells; GAE, gallic acid equivalent; QE, quercetin equivalent; IC_50_, Inhibitory concentration 50; EC_50_, Effective concentration 50; LC_50_, lethal dose 50; SRB, Sulforhodamine B.

## Sample Availability

Sample of the extracts are not available from the authors.
